# Testing for overall and cluster convergence of housing rents using robust methodology: evidence from Polish provincial capitals

**DOI:** 10.1007/s00181-021-02080-w

**Published:** 2021-06-06

**Authors:** Mateusz Tomal

**Affiliations:** Department of Real Estate and Investment Economics, Cracow University of Economics, Rakowicka 27, 31-510 Cracow, Poland

**Keywords:** Rental prices, Housing market, Convergence club, Stochastic convergence, Weak σ-convergence, Boosted Hodrick–Prescott, O18, R11, R21, R31, R52

## Abstract

The aim of this paper is to test for overall and cluster convergence of housing rents across Polish provincial capitals and to identify drivers of convergence club formation. In order to achieve the goal of the study, several novel convergence tests were used, including the Kong et al. (J Econom 209:185–207, 2019. https://doi.org/10.1016/j.jeconom.2018.12.022) and Phillips and Sul (Econometrica 75:1771–1855, 2007. https://doi.org/10.1111/j.1468-0262.2007.00811.x) approaches. Moreover, club convergence analysis was carried out in four different configurations, varying in the technique of trend component extraction from the data. In particular, three well-known methods of time series decomposition were used, i.e. the Hodrick–Prescott, Butterworth and Christiano–Fitzgerald filters, as well as the most recent boosted Hodrick–Prescott filter. The results indicated that rental prices across the studied cities do not share a common path in the long run. It is possible, however, to identify convergence clubs where rents are moving towards a club-specific steady state. Detailed analysis of the structure of estimated clusters showed that data filtering using the boosted Hodrick–Prescott method leads to the most reliable allocation of cities to convergence clubs. Moreover, the estimation of logit models revealed that the likelihood of any two cities belonging to the same convergence club depends mainly on similar levels in terms of the unemployment rate, housing stock, city area, and the number of students. Finally, recommendations for local and national policy-makers concerning the development of the rental market have been formulated, particularly in the areas of urban land-use planning policy, housing legislation and public–private partnerships.

## Introduction

Understanding price dynamics in the housing market is crucial both for private entities, such as real estate developers, but also for policy-makers, who can thus shape effective housing policies. The need for a proper understanding of the mechanisms of the housing market also stems from its huge impact on a country's economy, as was recognised in the recent global financial crisis.

One aspect of housing market dynamics is the phenomenon of price convergence or divergence. In the literature, a number of arguments can be found for the possibility of the convergence of sales/rental prices between spatially separated residential markets. In particular, as Apergis et al. (2015) point out, price convergence may be a consequence of the so-called ripple effect, which postulates the easy transfer of price shocks between property markets, due to existing population, labour market and financial capital mobility. It should be stressed that the occurrence of the ripple effect in the housing market has been empirically confirmed by studies conducted by Tomal (2020a) and Brzezicka et al. (2019), among others.

Conversely, however, assets in the housing market are highly heterogeneous and immobile (Małkowska et al. 2019), which may encourage price divergence. It is, therefore, difficult to expect that all local/regional housing markets in a given area will converge to a single equilibrium. A much more likely phenomenon is the occurrence of convergence clubs, i.e. the existence of several steady states to which prices are heading in the long term. The identification of price convergence clubs in the housing market is possible using a method developed by Phillips and Sul (2007, 2009). The empirical research to date in this area is not very extensive, and to the best of the author's knowledge only several such analyses have been developed. In particular, studies on the presence of convergence clubs in the residential market have been conducted in the USA (Kim and Rous 2012; Apergis and Payne 2012, 2019, 2020; Montañés and Olmos 2013), China (Meng et al. 2015; Lin et al. 2015; Zhang et al. 2017; Hu et al. 2020), the UK (Montagnoli and Nagayasu 2015; Holmes et al. 2019), Spain (Blanco et al. 2016), Australia (Awaworyi Churchill et al. 2018), South Africa (Apergis et al. 2015) and Poland (Matysiak and Olszewski 2019; Tomal 2019a, 2020a). It should be noted that almost all of the above studies analysed the convergence of sales prices in the residential market. The only exception was a study conducted by Zhang et al. (2017), in which the authors undertook price-to-rent ratio modelling. On the basis of the above literature review, a very clear research gap can therefore be observed, i.e. there are no analyses concerning the identification of housing rental convergence clubs. In general, residential rents are extremely poorly examined in terms of convergence. One of the very few studies on the subject was carried out by Bilgin et al. (2010), who analysed the stochastic convergence of housing rents between cities in Turkey.

Such a significant omission of the rental market in the analyses of the convergence phenomenon seems unjustified. This is due to the fact that it is the rental market, compared to home-ownership, that is currently developing very fast all over the world, in response to increasing property prices and the problem of affordability. In addition, as Czerniak and Rubaszek (2018) and Rubaszek and Rubio (2020) state, a well-developed rental market contributes to the reduction of fluctuations in the entire housing segment, as well as to macroeconomic stability. Therefore, the first objective of this article is to examine the processes of convergence of residential rents across Polish provincial capitals. This goal will be pursued in two stages. The first will assess the occurrence of overall convergence across the studied cities. In this case, four types of convergence will be examined, i.e. *β*-, *σ*-, stochastic and relative convergence. For the latter, the research method will be the Phillips and Sul (2007, 2009) approach, which will also be used in the second stage of the research aimed at testing for cluster convergence. The results of the study in the field of cluster convergence will enable the identification of clubs, in which rents tend to move towards a club-specific steady state in the long term.

Given that long-term price behaviour is the subject of the study, it should be noted that the calculations using the methodology outlined by Phillips and Sul (2007, 2009) should not be based on raw time series data but on an extracted trend component. Surveys to date have analysed convergence in the housing market based on only one selected filtration technique. The vast majority of papers are dominated by the Hodrick and Prescott (1997) filter, with the exception of a study conducted by Holmes et al. (2019), who used the Hamilton (2018) filter. The second objective of this study was formulated on this basis and is to compare the club convergence analysis estimates obtained based on various time series filtering techniques. In particular, the comparison will include an analysis of the structure of the estimated convergence clubs, as well as the dynamics of their establishment over subsequent 3-year periods. In addition, using a logit model, the drivers for the formation of the convergence clubs will be checked.

As mentioned above, currently, the rental market is becoming an extremely important part of the real estate sector and complementary to home-ownership in Poland as well (Tomal 2020b). It is, therefore, important to analyse the relationship between sales and the rental housing market. Consequently, the third objective of this analysis is to compare the results of the identified convergence clubs in the rental market with those found in the sales market. The implementation of this goal is also important from the point of view of formulating potential recommendations for policy-makers regarding the development of the residential rental market in Poland.

In summary, this article aims to answer the following research questions:

1. Do residential rents across the surveyed rental markets converge to one steady state in the long term? If not, can convergence clubs be identified among the cities studied?
2. In the case of absence of overall convergence in the Polish rental market, do club convergence analysis estimates differ depending on the time series filtering technique? Is the number of convergence clubs stable over time? Is the estimated structure of convergence clubs the same as in the case of the residential sales market?
3. In the case of absence of overall convergence in the Polish rental market, which factors drive the formation of the identified convergence clubs?

This study contributes to the existing literature in several ways. Firstly, this is the first paper that identifies convergence clubs in the residential rental market. Secondly, this work is unique because of the use of several different filtering methods to extract the trend component from the data. In particular, the Hodrick and Prescott (1997), Butterworth (1930), Christiano and Fitzgerald (2003) filters, as well as the very recently developed boosted Hodrick–Prescott filter developed by Phillips and Shi (2020) will be used. Finally, a completely new approach outlined by Kong et al. (2019) will be employed to assess the occurrence of *σ*-convergence.

The rest of the article is organised as follows. Section 2 describes the data and provides a detailed overview of the convergence test methods. Section 3 contains the results of the analysis and their discussion. The final Sect. 4 presents the main conclusions of the study, as well as recommendations for local and national policy-makers concerning the development of the rental market, particularly in the areas of urban land-use planning policy, housing legislation and public–private partnerships.

## Methodology and data

### Data and study area

The data used in this study were obtained from the National Bank of Poland (https://www.nbp.pl/). At the moment, this is the most detailed source of information on housing rents in Poland. It should also be emphasised that the analysed data concern only the secondary market, as the primary residential rental market in Poland practically does not exist.

**Fig. 1:**
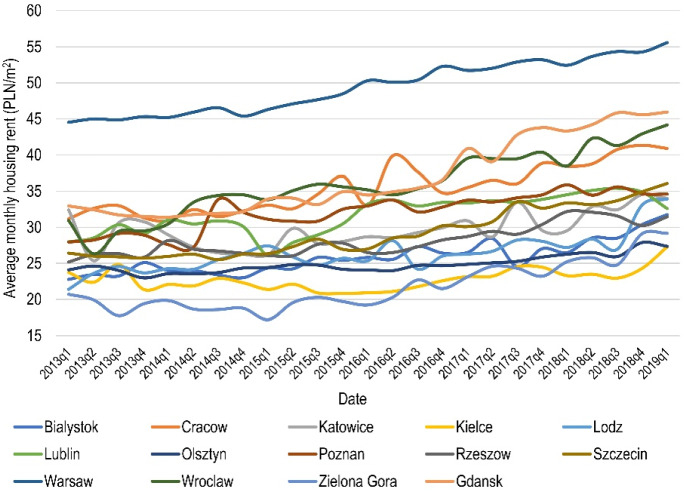
Temporal dynamics of average monthly housing rents (PLN/m^2^) in the cities

The time series used in this study covers the period 2013q1–2019q1 and represents the average monthly nominal housing rent per square meter in each Polish provincial capital.^1^ Two cities, however, i.e. Opole and Bydgoszcz, were omitted from the study due to lack of data for the entire analysed time frame. Figure 1 presents the dynamics of rental prices in the surveyed cities, on the basis of which it can be concluded that, by far, the highest rents are in the Polish capital, i.e. Warsaw. Other cities, which differ significantly from the rest in terms of rental price levels, are Cracow, Wroclaw and Gdansk.

It should be noted that Fig. 1 shows raw data, which have not been processed in any way. During convergence tests, a seasonally adjusted time series containing information on housing rentals in natural log form will be used. In addition, in order to perform the convergence test using the log *t* regression method, a trend component will be extracted from the time series described above, which is detailed in the next subsection.

### Testing for overall convergence

In order to examine the overall convergence of housing rents in Polish provincial capitals, four research methods will be used to assess the occurrence of *β*-, *σ*-, stochastic, and relative convergence. In the context of this article, *β*-convergence assumes that cities with low residential rents at the beginning of the analysed period are, at the same time, characterised by a high average rental price growth rate. This type of convergence can be examined using the following equation:

1 $$\frac{{\ln \left( {\frac{{p_{iT} }}{{p_{i0} }}} \right)}}{T} = \alpha + \beta \ln p_{i0} + \varepsilon_{i}$$

where $p_{iT}$ is the average rental price in city $i \left( {i = 1, \ldots ,N} \right)$ at time $T \left( {t = 1, \ldots ,T} \right)$, $p_{i0}$ denotes the initial average housing rent in city $i$, $\alpha$ represents the constant term, $\varepsilon_{i}$ is the error term, and $\beta$ is the coefficient. If, following the estimation of the above model, the fitted slope coefficient ($\hat{\beta }$) is statistically significant and has a negative value, then the phenomenon of unconditional convergence between cities can be said to exist. Analysing convergence by means of Eq. (1) examines its absolute version. If the above equation is supplemented by a set of covariates, conditional convergence is tested. It should be noted, however, that *β*-convergence has several disadvantages. First of all, the fitted slope coefficient is biased due to the endogeneity problem (Bai et al. 2019). Secondly, Eq. (1) is cross-sectional and does not take into account price changes over time. Finally, *β*-convergence does not provide information on whether or not there is a process of price equalisation between locations. In order to assess the occurrence of the latter phenomenon, the concept of *σ*-convergence should be used. It should also be stressed that there is a relationship between *β*- and *σ*-convergence in the sense that the former is necessary for the latter to appear (Young et al. 2008).

Until now, there has been no robust econometric framework to study *σ*-convergence. Recently, however, Kong et al. (2019) developed a very powerful tool for studying this type of convergence (hereinafter referred to as the KPS approach). This new methodology uses the following trend regression to verify the occurrence of *σ*-convergence:

2 $$S_{t} = \alpha + \phi t + \varepsilon_{t}$$

where $S_{t} = \frac{1}{N}\mathop \sum \nolimits_{i = 1}^{N} \left( {\ln p_{it} - \overline{\ln p}_{t} } \right)^{2}$ and denotes the cross-sectional variance of log level rental prices at time $t$, $\overline{{{\text{ln}}p}}_{t}$ is the cross-sectional mean of log level rental prices at time $t$, $\varepsilon_{t}$ is the error term, and $\phi$ represents the slope coefficient. In the KPS method, *σ*-convergence is present if the fitted slope coefficient $\hat{\phi }$ is negative and statistically significant. The assessment of the significance of $\hat{\phi }$ is based on a comparison of the estimated robust *t*-statistic $\left( {t_{{\hat{\phi }}} } \right)$ to a critical value of ± 1.65. In particular, if $t_{{\hat{\phi }}} < - 1.65$ there is convergence; however, if $t_{{\hat{\phi }}} > 1.65$, there is divergence. The advantage of the KPS approach is that it allows for: (1) misspecification of Eq. (2); and (2) serially correlated and heteroskedastic residuals. It should also be noted that for the empirical application of the method described above, the lag truncation parameter ($L$) should be determined.^2^ Following Kong et al. (2019), the calculations in this study will be made for $L = \left\{ {1,3,5,7} \right\}$.

Another type of convergence that will be examined in this article is the so-called stochastic convergence, which in a panel setting can be defined as (Próchniak and Witkowski 2016):

3 $$\mathop {\lim }\limits_{k \to \infty } \left( {{\text{ln}}p_{i,t + k} - \overline{{{\text{ln}}p}}_{t + k} |I_{t} } \right) = 0$$

where $I_{t}$ denotes the set of information at time $t$, while the rest of the markings are as above. In order to examine empirically whether there is stochastic convergence, the stationarity of the following time series should be checked:

4 $$y_{it} = {\text{ln}}p_{it} - \overline{{{\text{ln}}p}}_{t} = {\text{ln}}\left( {\frac{{p_{it} }}{{\overline{p}_{t} }}} \right)$$

It should be stressed that in the data used in this article cross-sectional dependence was identified using the Pesaran (2004) and Breusch and Pagan (1980) tests; therefore, the selected unit root test must be resistant to this type of phenomenon. On this basis, this study will apply the cross-sectionally augmented DF (CADF) test outlined by Pesaran (2007a). The idea of the above method is to extend the DF test with a cross-sectional average of: (1) the variable defined above with a time lag of one period, as well as (2) its first difference. Taking this into account, the CADF regression^3^ in the context of this article is as follows:

5 $$\Delta y_{it} = \alpha_{i} + \rho_{i} y_{i,t - 1} + \varphi_{i} \overline{y}_{t - 1} + \vartheta_{i} \Delta \overline{y}_{t} + \varepsilon_{it}$$

where $\rho_{i}$, $\varphi_{i}$, $\vartheta_{i}$ are parameters to be estimated for each studied city separately. Testing for the unit root consists in checking the statistical significance of the fitted parameter for a lagged variable. In particular, if the estimated *t*-statistic for $\hat{\rho }_{i}$ ($t_{{\hat{p}_{i} }}$—known also as CADF_*i*_) is less than the critical value, the unit root hypothesis is rejected, which means that stochastic convergence takes place. Using the calculated values of CADF_*i*_, it is also possible to check the panel stationarity as a whole. For this purpose, the CIPS (cross-sectionally augmented Im, Pesaran and Shin) statistic, as an arithmetic mean of CADF_*i*_, must be calculated. Critical values for the CADF and CIPS tests are presented for the different significance levels in Pesaran (2007a). It should also be noted that Eq. (5) is able to indicate whether there is a process of equalisation of rental prices between a given city and the Polish average to a constant value or to zero. For the latter to occur, both the estimated *t*-statistic for $\hat{\rho }_{i}$ must be less than the critical value and the fitted parameter $\hat{\alpha }_{i}$ must be statistically insignificant.

Finally, the concept of relative convergence introduced by Phillips and Sul (2007, 2009) will also be used to assess overall convergence in this study. Generally speaking, the two time series converge relatively over time if their ratio is heading towards unity in the long term. The approach outlined by Phillips and Sul (2007, 2009) (hereinafter referred to as the PS approach) is widely used in practice due to its advantages. First of all, the PS approach takes into account the heterogeneity of the examined units and its change over time. Secondly, the method does not depend on assumptions regarding trend stability or stochastic non-stationarity in the panel common component. Thirdly, this method overcomes the problem of the biased convergence parameter appearing at *β*-convergence. Fourthly, the PS approach is treated as an asymptotic cointegration test without suffering from the small sample problems.^4^ Fifthly, the analysis is based on a general form of a nonlinear time-varying factor model. Finally, in the absence of overall convergence between the units analysed, the PS approach allows the identification of convergence clubs (Apergis and Cooray 2014).

The starting point of the relative convergence examination using the PS approach in the context of this study is the following decomposition of the panel of average rental prices:

6 $${\text{ln}}p_{it} = \delta_{it} \mu_{t}$$

where $\delta_{it}$ is the changing over time idiosyncratic component measuring the relative share of city $i$ at time $t$ in $\mu_{t}$, i.e. in a common trend component. Further, $\delta_{it}$ can be modelled in a semiparametric form as:

7 $$\delta_{it} = \delta_{i} + \sigma_{i} \xi_{it} \log \left( t \right)^{ - 1} t^{ - \alpha }$$

where $\xi_{it} \sim iid\left( {0,1} \right)$ across $i$, $\alpha$ is a parameter indicating the speed of convergence, $\sigma_{i}$ is a scale parameter, $\log \left( t \right)$ denotes a slowly varying function, and $\delta_{i}$ is fixed. On this basis, the null hypothesis of convergence can be presented as follows:

8 $$\begin{array}{*{20}l} {H_{0} : \delta_{i} = \delta \;{\text{and}}\; \alpha \ge 0} \hfill & {\quad {\text{for convergence in rates}}} \hfill \\ {H_{0} : \delta_{i} = \delta \;{\text{and}}\;\alpha \ge 1} \hfill & {\quad {\text{for convergence in levels}}} \hfill \\ \end{array}$$

whereas the alternative hypothesis of divergence is:

9 $$H_{A} : \delta_{i} \ne \delta \;{\text{for}}\;{\text{all}}\; i\; {\text{or}}\; \alpha < 0$$

The first stage for the empirical verification of the hypotheses set out above is to estimate the cross-sectional variance according to the following formula:

10 $$H_{t} = \frac{1}{N}\mathop \sum \limits_{i = 1}^{N} \left( {h_{it} - 1} \right)^{2} ,\;h_{it} = \frac{{\ln p_{it} }}{{N^{ - 1} \mathop \sum \nolimits_{i = 1}^{N} \ln p_{it} }}$$

where $h_{it}$ is the transition path of city *i* at time *t*, which represents the ratio of log level rental price in city *i* and panel average at time *t*. In case $h_{it} \to 1$ for all *i*, as $t \to \infty$, or when $H_{t} \to 0$, as $t \to \infty$, there is a convergence process. The next stage of the convergence study according to the PS approach is the estimation of the so-called log *t* regression, which is given by formula^5^:

11 $$\log \left( {\frac{{H_{1} }}{{H_{t} }}} \right) - 2\log \left( {\log \left( t \right)} \right) = a + b\log \left( t \right) + \varepsilon_{t}$$

where $t = \left[ {rT} \right],\left[ {rT} \right] + 1, \ldots ,T$ for $r \in \left[ {0.2,0.3} \right]$ and ${b}$ is the regression parameter. In particular, if $\hat{b}$ is significantly smaller than zero, there is a process of divergence, i.e. all cities do not share a common trend in the long run. Conversely, in the case of $\hat{b} \ge 2$, there is a convergence in levels, which means that the rental prices from the surveyed cities aim at one common value. Finally, if $2 > \hat{b} \ge 0$, there is growth convergence, which indicates that housing rent growth rates across cities tend to converge. The significance of parameter $\hat{b}$ in the PS approach is usually assessed at a level of 0.05, for which the critical value is − 1.65. An important element of the log *t* regression is also the selection of the sample fraction (*r*) to be excluded from the estimation. For a short time series, i.e. when $T \le 50$, the setting $r = 0.3$ is suggested (Phillips and Sul 2007).^6^

In the context of the analysis of rental price convergence before applying the PS approach, the cycle component should be removed from each time series because it improves the finite sample power and size of the test (Phillips and Sul 2007). When the subject of the analysis is a raw rental price time series, Eq. (6) concerning panel data decomposition is as follows:

12 $${\text{ln}}p_{it} = \delta_{it} \mu_{t} + \tau_{it}$$

where $\tau_{it}$ is a business cycle component for city *i* at time *t*.

In this article, four filtering techniques were used to extract the trend component from the data. The first is the well-known Hodrick and Prescott (HP) (1997) filter, for which, due to the quarterly nature of the data, a tuning parameter ($\lambda$) equal to 1600 was set. The above filter, however, has been criticised in recent years, among other things because of the problem of selecting the optimal value of the $\lambda$ parameter, which has a significant impact on the result of the filtration. As noted by Phillips and Shi (2020), if $\lambda$ is too large, the HP fitted trend creates a residual trend, which pollutes the cyclical component. If $\lambda$ is too small, however, the fitted trend is too flexible and imitates short-term fluctuations. Therefore, Phillips and Shi (2020) developed a modification of the standard HP filter, which they called boosted HP filter. This method is based on checking whether the cyclical component contains elements of the trend after using the standard HP filter. If so, the HP filter is reapplied to it to remove leftover trend residuals. When a new cyclic component is also characterised by trending behaviour, the whole process is repeated and continues until the trend elements are completely removed. As a criterion for stopping the iteration process, the ADF test can be used, which examines after which time the cyclic component is stationary (assumed significance level 0.05). In addition to the HP and boosted HP methods, the Butterworth (1930) and Christiano and Fitzgerald (CF) (2003) filters were used in this study.

### Testing for cluster convergence

It should be noted that in the absence of overall convergence across the studied cities, it is possible to identify so-called convergence clubs, in which the rental prices of members of a given cluster head towards a club-specific steady state. The PS approach described above will be used to assess the occurrence of convergence clubs.^7^ Under this method, the identification of city clusters takes place in the following steps (Du 2017):

1. arranging the cities in the panel in decreasing order according to housing rents in the last period;
2. forming a core group of cities $k^{*}$ on the basis of the log *t* test maximising $t_{k}$, subject to $t_{k} > - 1.65$, starting with the *k* highest individuals in the panel, where $N > k \ge 2$;
3. adding one city to the core group formation at a time and running the log *t* regression. If the obtained *t*-statistic is above the critical value (*c*) equal to 0 (for small *T*), include the city in the core group. Repeat this procedure for the rest of the cities. At the end, run the log *t* test for the extended core group of cities and make sure that the calculated *t*-statistic is greater than − 1.65. If so, the first convergence club is created. If not, repeat step 3 but firstly raise the critical value *c*;
4. for the remaining cities, forming a potential new convergence club and running the log *t* regression. If the obtained *t*-statistic is above − 1.65, end the whole clustering procedure. Otherwise, repeat steps 1–3 to check if the remaining subgroup of cities can be further subdivided into convergence clubs;
5. running a club merging procedure to check if the convergence clubs created in steps 1–4 can be combined. In this paper, the club merging will be conducted subsequently. According to this approach, the log *t* test is run for all subsequent pairs of the initial clubs. If the calculated *t*-statistic for a pair indicates that there is a convergence, merge the clubs from this pair into one club.

## Results

### Overall convergence estimates

**Fig. 2:**
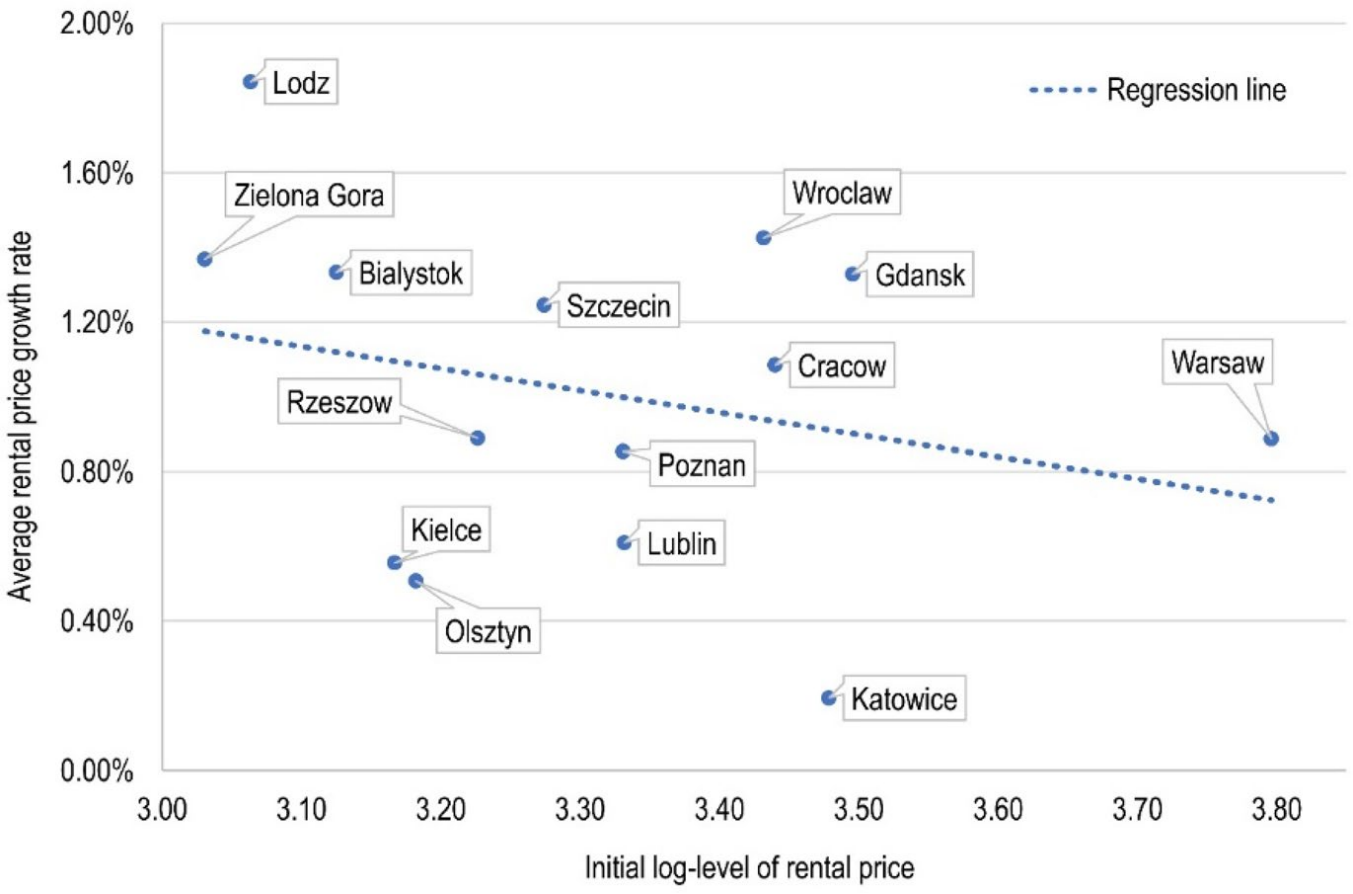
Visualisation of *β*-convergence test estimates

The first stage of the empirical study was the assessment of overall convergence based on the *β*-convergence concept. The results of Eq. (1) revealed a negative slope coefficient value ($\hat{\beta } = {-}\,0.0059$). The convergence parameter, however, did not turn out to be significant even at the 0.10 level. Therefore, taking into account the whole panel, it is not possible to identify the occurrence of a phenomenon in which cities with low initial rental prices are simultaneously characterised by high average growth rate over the analysed period. Detailed estimates of the *β*-convergence test are presented in Fig. 2, from which it can be concluded that lack of *β*-convergence is mainly caused by cities such as Katowice and Wroclaw, which are characterised by a very similar initial price but a diametrically different growth rate. Figure 2 also provides the information that, for the surveyed cities, the average rental price growth rate ranges from approx. 0.2% to almost 2% in the case of the city of Lodz, which at the same time has almost the lowest initial rental price level.

**Fig. 3:**
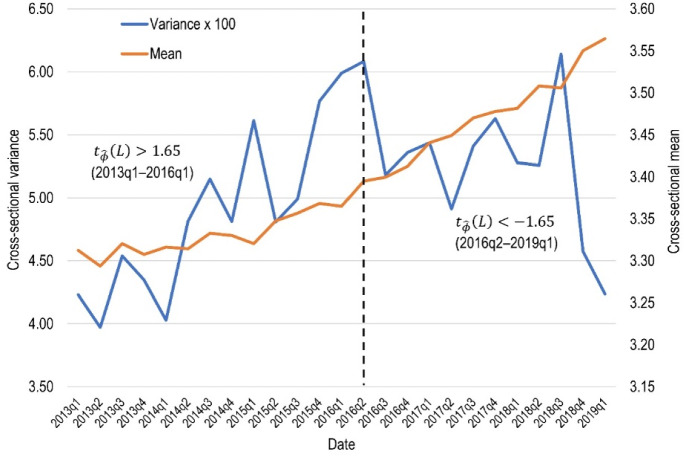
Visualisation of *σ*-convergence test estimates

Then, the occurrence of *σ*-convergence was checked using the KPS approach. Estimates obtained using the above-mentioned method, similarly to *β*-convergence, indicated the lack of *σ*-convergence in the examined period (Table 1). This means that rental prices across the studied cities do not show a tendency to equalise. When analysing the cross-sectional variance of housing rents in Fig. 3, however, it can be seen that from the second quarter of 2016, the variance of rental prices is decreasing. Therefore, the *σ*-convergence study was also conducted in two sub-periods: (1) 2013q1–2016q1; and (2) 2016q2–2019q1. The results showed that for the first 3 years there is a process of quite strong divergence, while for the second sub-period a convergence of rental prices can be observed (independently of the lag truncation parameter). Such a result may be related to the housing sales prices, which by 2016 were stagnant or slightly increasing, but after that time began to grow dramatically. This may have resulted in an increased demand for renting in relation to home-ownership, which could have accelerated the convergence process, as rent is a much more flexible form of housing and, therefore, prices can be "transferred" much quicker between locations.

**Table 1:** The KPS test results for *σ*-convergence

Period	$\hat{\phi }$	$t_{{\hat{\phi }}} \left( 1 \right)$	$t_{{\hat{\phi }}} \left( 3 \right)$	$t_{{\hat{\phi }}} \left( 5 \right)$	$t_{{\hat{\phi }}} \left( 7 \right)$	Conclusion
Whole: 2013q1–2019q1	0.00036*	2.34	2.05	1.93	1.85	Divergence
First 3 years: 2013q1–2016q1	0.00132*	6.94	8.73	12.67	11.73	Divergence
Last 3 years: 2016q2–2019q1	− 0.00065*	− 1.91	− 1.90	− 2.06	− 2.22	Convergence

*Significant at 0.05 level

Another surveyed type of convergence in the Polish residential rental market was stochastic convergence. The results of this analysis are presented in Table 2. In particular, the CADF unit root test estimates indicate that only four out of the 14 examined cities stochastically converge to the average rental price in Poland. Moreover, the verification of the statistical significance of parameter $\hat{\alpha }_{i}$ shows that rental prices in Katowice and Lodz are heading towards exactly the same level as the panel average. Conversely, between rents in Cracow and Bialystok and the Polish average rental price, there is a convergence to nonzero constant (positive for Cracow and negative for Bialystok).

**Table 2:** Estimates of stochastic convergence using the CIPS and CADF unit root tests

City	$\hat{\rho }_{i}$	$\widehat{{{\text{CADF}}}}_{i}$	$\hat{\alpha }_{i}$	Conclusion
Bialystok	− 1.0354*	− 4.7882	− 0.1582*	Convergence to a nonzero constant
Katowice	− 0.9112*	− 4.9631	− 0.0133	Convergence to zero
Kielce	− 0.3014	− 2.2895	− 0.0870	Divergence
Cracow	− 0.9873*	− 4.4287	0.1603*	Convergence to a nonzero constant
Lublin	− 0.3115	− 1.4674	0.0168	Divergence
Lodz	− 0.6384*	− 3.0928	− 0.0740	Convergence to zero
Olsztyn	− 0.1185	− 1.0625	− 0.0303	Divergence
Poznan	− 0.5631	− 2.6409	0.0433	Divergence
Rzeszow	− 0.5322	− 2.5416	− 0.0353	Divergence
Szczecin	− 0.2707	− 1.5828	− 0.0069	Divergence
Gdansk	− 0.1403	− 1.1984	0.0333	Divergence
Warsaw	− 0.4170	− 2.0336	0.2077	Divergence
Wroclaw	− 0.2541	− 1.7137	0.0491	Divergence
Zielona Gora	− 0.3159	− 1.6566	− 0.1059	Divergence
Whole panel	N/A	− 2.5329*^‡^	N/A	Some time series in the panel are stationary

The convergence benchmark is the average rental price in Poland

*N/A* not applicable

*Significant at 0.05 level

^‡^Represents the CIPS test statistics value

**Fig. 4:**
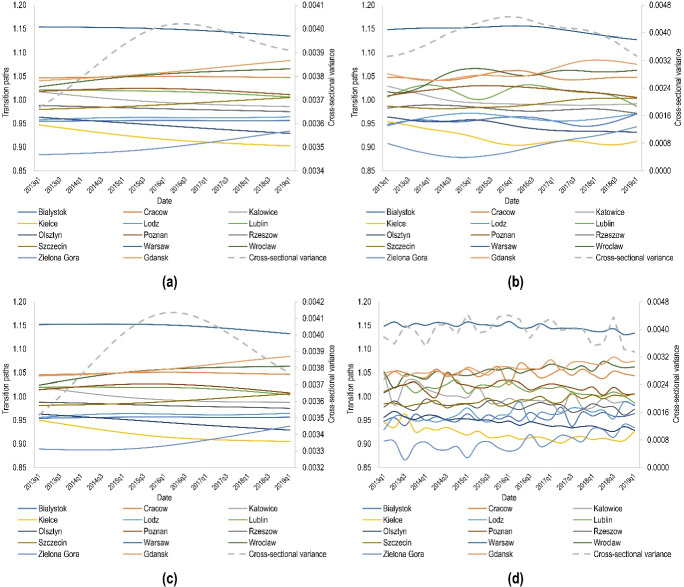
Transition paths and cross-sectional variance for the cities calculated based on the trend component of time series extracted using **a** the Hodrick–Prescott filter, **b** the boosted Hodrick–Prescott filter, **c** the Butterworth filter and **d** the Christiano–Fitzgerald filter

The final method to study the overall convergence of housing rents across Polish provincial capitals was the log *t* regression. As with previous tests, the results obtained with the PS approach also indicated that the residential rental markets surveyed do not share the common trend in the long term, as shown by the $t_{{\hat{b}}}$ values, which regardless of the data filtering method, are well below the critical value of − 1.65 (Table 3). Moreover, on the basis of transition paths analysis (Fig. 4a–d), a very strong heterogeneity can be observed among the analysed cities both in the cross-sectional and temporal dimensions. In particular, in the case of data filtering with the help of the HP, boosted HP and Butterworth filters, until 2016, it is clear that rental prices tend to diverge. In contrast, in the subsequent period, transition paths are beginning to strive for unity. Similarly, the cross-sectional variance marked in Fig. 4a–d also decreases from 2016 onwards, which confirms the convergence phenomenon. It should be noted that the above conclusions are consistent with those drawn from the KPS test. Furthermore, it can be noted that filtering time series of rental prices using the CF method largely fails to get rid of the cyclical component, which may be due to the fact that this filter is optimal only for data that are generated by pure random walk, while for other time series describing, for example, inflation, interest rates or unemployment this method is nearly optimal^8^ (Christiano and Fitzgerald 2003).

**Table 3:** Log *t* regression test estimates for the whole sample depending on the filtering technique used to extract the trend component of time series

Filter	$\hat{b}$	$t_{{\hat{b}}}$	Conclusion
Hodrick–Prescott	− 0.7269*	− 35.2626	Divergence
boosted Hodrick–Prescott	− 0.5338*	− 10.7015	Divergence
Butterworth	− 0.6692*	− 22.4372	Divergence
Christiano–Fitzgerald	− 0.5719*	− 8.7975	Divergence

*Significant at 0.05 level

**Fig. 5:**
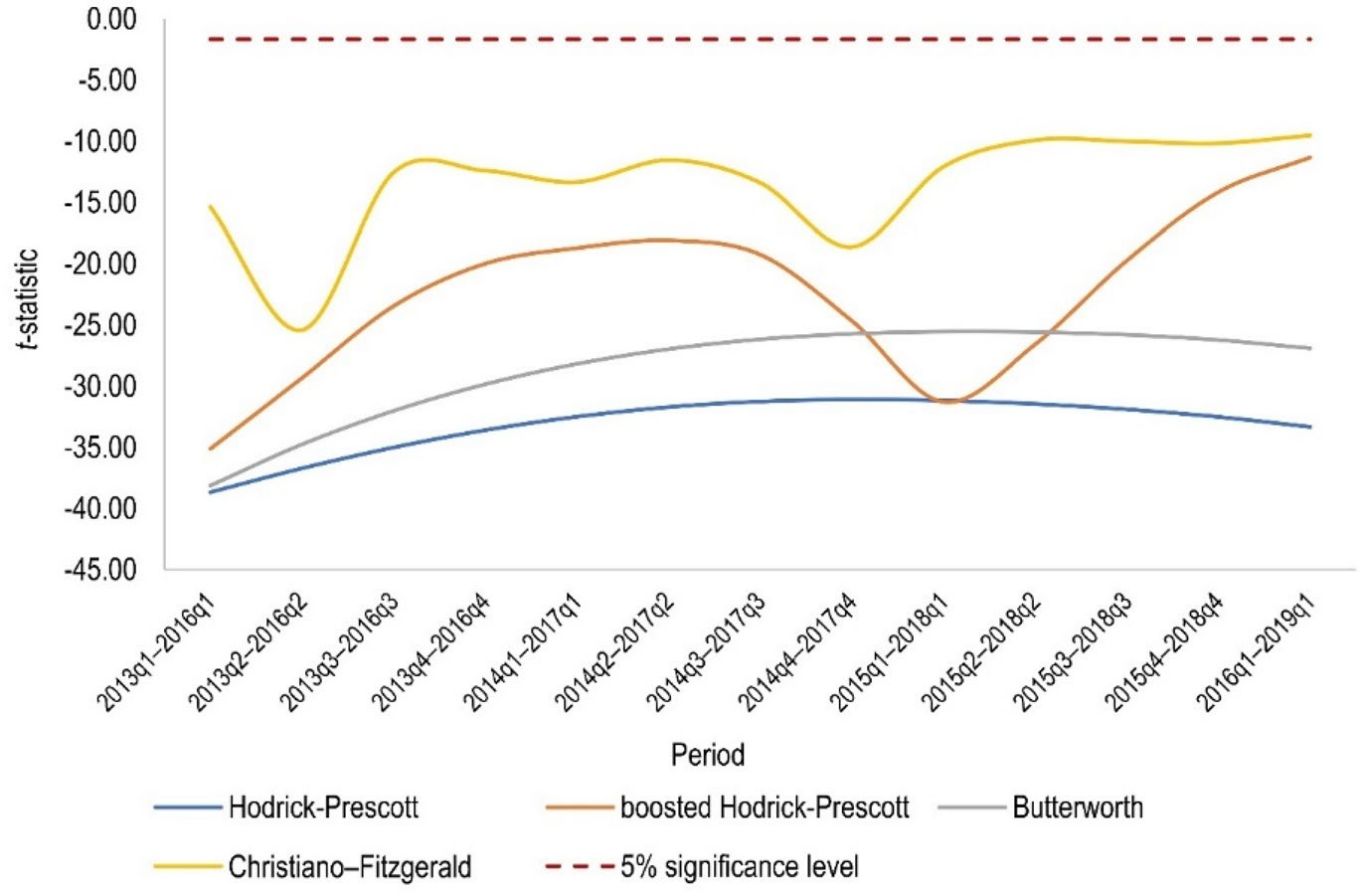
The value of the *t*-statistic from the log *t* regression estimates in subsequent 3-year periods depending on the data filtering method

It should be stressed that according to the log *t* test, overall convergence of rental prices does not exist for different test periods as well. In particular, Fig. 5 presents *t*-statistics of the log *t* regression for subsequent 3-year periods. It can be seen that all lines in Fig. 5 are significantly below the critical value. It can only be pointed out that the *t*-statistic estimates dynamics is very similar for data filtration according to the HP and Butterworth filters.^9^

Summarising the overall convergence analysis, it should be stressed that all research methods used in this study rejected the possibility of its occurrence across the Polish residential rental markets, which is in line with other analyses studying convergence or differentiation in the Polish real estate market; see, for example, Żelazowski (2019), Gnat (2016), Dittmann (2014), Wiśniewski and Brzezicka (2020), Tomal (2019a), Tomal and Gumieniak (2020).

It seems that lack of overall convergence in the rental market is to be expected. Firstly, as mentioned earlier, housing goods are heterogeneous and immobile. Moreover, lack of convergence of rental prices among Polish cities results also from the exceptionally uneven and poorly developed rental market in Poland. In particular, according to Eurostat (2020) and OECD (2020), the share of households renting on market terms hovers around 5%, and most of the supply of housing on the rental market is offered in Cracow, Warsaw, Wroclaw and Gdansk. In addition, prices on the long-term rental market in Poland are very much distorted by short-term rentals, which until now have not been regulated to any extent. Moreover, the rental market in Polish cities is influenced by the number of students, which causes the number of units for long-term rent to decrease. In addition, the so-called institutional housing lease market, which is very well developed in other European countries, is hardly functioning in Poland. The specificity of the Polish residential rental market described above fully justifies the lack of overall convergence and, also, gives rise to the assumption that individual cities may form convergence clubs where rental prices are heading towards a club-specific steady state.

### Cluster convergence estimates

Table 4 presents preliminary results of the allocation of the examined cities to convergence clubs. In the case of data filtering with the HP and Butterworth filters, the divergent cases are also visible where rental prices tend to move towards a city-specific steady state. When analysing in detail the division of the surveyed cities into clusters, it can be observed that in the case of cities such as Gdansk, Wroclaw, Warsaw, Szczecin and Poznan there are no differences between club clustering procedure estimates. For the remaining cities, depending on the filter used, we can see quite a significant variation in terms of belonging to a convergence club.

**Table 4:** Club convergence analysis estimates: initial classification

City	Initial club classification depending on filtering method			
	Hodrick–Prescott	Boosted Hodrick–Prescott	Butterworth	Christiano–Fitzgerald
Bialystok	*Club 3*	*Club 3*	Group	*Club 3*
Katowice	**Club 2**	**Club 2**	**Club 2**	*Club 3*
Kielce	Group	Club 4	Group	Club 4
Cracow	Group	***Club 1***	Group	**Club 2**
Lublin	**Club 2**	**Club 2**	**Club 2**	*Club 3*
Lodz	*Club 3*	*Club 3*	Group	*Club 3*
Olsztyn	Group	Club 4	Group	Club 4
Poznan	**Club 2**	**Club 2**	**Club 2**	**Club 2**
Rzeszow	*Club 3*	**Club 2**	**Club 2**	*Club 3*
Szczecin	**Club 2**	**Club 2**	**Club 2**	**Club 2**
Gdansk	***Club 1***	***Club 1***	***Club 1***	***Club 1***
Warsaw	***Club 1***	***Club 1***	***Club 1***	***Club 1***
Wroclaw	***Club 1***	***Club 1***	***Club 1***	***Club 1***
Zielona Gora	**Club 2**	***Club 1***	**Club 2**	**Club 2**
Log *t* regression estimates				
$\hat{b}\left( {club 1} \right)$	0.2619	0.0256	0.2971	0.2282
$\hat{b}\left( {club 2} \right)$	0.3021	0.1649	0.4478	0.1885
$\hat{b}\left( {club 3} \right)$	0.0023	− 0.3443	N/A	0.4713
$\hat{b}\left( {club 4} \right)$	N/A	0.5744	N/A	1.1103
$\hat{b}\left( {group} \right)$	− 1.0897*	N/A	− 0.9799*	N/A
$t_{{\hat{b}}} \left( {club 1} \right)$	4.1886	0.3659	4.2038	2.8123
$t_{{\hat{b}}} \left( {club 2} \right)$	3.7982	0.6794	4.4013	1.0348
$t_{{\hat{b}}} \left( {club 3} \right)$	0.0612	− 0.1286	N/A	1.0841
$t_{{\hat{b}}} \left( {club 4} \right)$	N/A	2.4532	N/A	1.2548
$t_{{\hat{b}}} \left( {group} \right)$	− 56.9367	N/A	− 33.8944	N/A
Club merging test estimates				
$\hat{b}\left( {clubs: 1 + 2} \right)$	− 0.4213*	− 0.2139*	− 0.4055*	− 0.2164*
$\hat{b}\left( {clubs: 2 + 3} \right)$	0.1272	− 0.4555*	N/A	0.1633
$\hat{b}\left( {clubs: 3 + 4} \right)$	N/A	− 0.7849*	N/A	− 0.5475*
$t_{{\hat{b}}} \left( {clubs: 1 + 2} \right)$	− 12.4733	− 3.5267	− 9.6475	− 2.2428
$t_{{\hat{b}}} \left( {clubs: 2 + 3} \right)$	1.9349	− 1.7037	N/A	0.8004
$t_{{\hat{b}}} \left( {clubs: 3 + 4} \right)$	N/A	− 5.4025	N/A	− 3.2706

Cities in the group are divergent cases and do not share a common path in the long run

*N/A* not applicable

*Significant at 0.05 level

After the initial assignment of cities to convergence clubs, it was checked whether some city clusters could be merged. In particular, as shown in Table 4, this is only possible for the initial clubs 2 and 3 ($t_{{\hat{b}}} > - \,1.65$) when data are filtered with the HP and CF filters. After the merging procedure, the final classification of cities to convergence clubs is presented in Table 5. The final results of the cluster convergence analysis reveal that for eight out of the 14 examined cities, i.e. Wroclaw, Warsaw, Gdansk, Szczecin, Rzeszow, Poznan, Lublin and Katowice, the obtained allocation does not change depending on the data filtering method. To the contrary, small differences in assignment are observed for the city of Zielona Gora. For the rest of the cities, there are quite large discrepancies in terms of club membership. Furthermore, from Table 5 it can be concluded that the most similar club convergence analysis estimates are observed when applying the HP, Butterworth and CF filters (9–12 compatible matches). The results of the cluster procedure based on the boosted HP filter, however, are only in line with those obtained from other data filtering methods in the case of eight cities. Moreover, this filter makes by far for the largest fragmentation of the Polish rental market by creating as many as four convergence clubs. Cluster convergence estimates obtained from data extraction with the boosted HP filter, however, seem by far to be the most reliable. In particular, only in this case is the city of Cracow assigned to club 1, which would be expected, as Cracow has the second largest residential rental market in Poland after Warsaw. Moreover, created clubs 3 and 4 seem to be exceptionally accurate, which is confirmed in Fig. 2, showing that rental prices in these cities are very similar in terms of initial rent and its growth rate. It should also be stressed that all identified convergence clubs are characterised only by convergence in rates, i.e. the cities in individual clubs do not tend to reach a certain price level, but share a common growth path in the long term.

**Table 5:** Club convergence analysis estimates: final classification

City	Final club classification depending on filtering method				
	Rental housing market (this study)				Sales secondary housing market classification according to Tomal (2019a, b) estimates
	Hodrick–Prescott	boosted Hodrick–Prescott	Butterworth	Christiano–Fitzgerald	Hodrick–Prescott
Bialystok	**Club 2**	*Club 3*	Group	**Club 2**	*Club 3*
Katowice	**Club 2**	**Club 2**	**Club 2**	**Club 2**	*Club 3*
Kielce	Group	Club 4	Group	*Club 3*	*Club 3*
Cracow	Group	***Club 1***	Group	**Club 2**	***Club 1***
Lublin	**Club 2**	**Club 2**	**Club 2**	**Club 2**	**Club 2**
Lodz	**Club 2**	*Club 3*	Group	**Club 2**	*Club 3*
Olsztyn	Group	Club 4	Group	*Club 3*	*Club 3*
Poznan	**Club 2**	**Club 2**	**Club 2**	**Club 2**	**Club 2**
Rzeszow	**Club 2**	**Club 2**	**Club 2**	**Club 2**	**Club 2**
Szczecin	**Club 2**	**Club 2**	**Club 2**	**Club 2**	*Club 3*
Gdansk	***Club 1***	***Club 1***	***Club 1***	***Club 1***	***Club 1***
Warsaw	***Club 1***	***Club 1***	***Club 1***	***Club 1***	***Club 1***
Wroclaw	***Club 1***	***Club 1***	***Club 1***	***Club 1***	**Club 2**
Zielona Gora	**Club 2**	***Club 1***	**Club 2**	**Club 2**	*Club 3*
Log *t* regression estimates					
$\hat{b}\left( {club 1} \right)$	0.2619	0.0256	0.2971	0.2282	0.130
$\hat{b}\left( {club 2} \right)$	0.1272	0.1649	0.4478	0.1307	0.244
$\hat{b}\left( {club 3} \right)$	N/A	− 0.3443	N/A	1.1103	0.083
$\hat{b}\left( {club 4} \right)$	N/A	0.5744	N/A	N/A	N/A
$\hat{b}\left( {group} \right)$	− 1.0897*	N/A	− 0.9799*	N/A	N/A
$t_{{\hat{b}}} \left( {club 1} \right)$	4.1886	0.3659	4.2038	2.8123	4.018
$t_{{\hat{b}}} \left( {club 2} \right)$	1.9349	0.6794	4.4013	0.8004	18.042
$t_{{\hat{b}}} \left( {club 3} \right)$	N/A	− 0.1286	N/A	1.2548	2.281
$t_{{\hat{b}}} \left( {club 4} \right)$	N/A	2.4532	N/A	N/A	N/A
$t_{{\hat{b}}} \left( {group} \right)$	− 56.9367	N/A	− 33.8944	N/A	N/A

Cities in the group are divergent cases and do not share a common path in the long run

Using the merging procedure proposed by Schnurbus et al. (2017), the division of cities into clubs is identical to that presented in the table above

*N/A* not applicable

*Significant at 0.05 level

Finally, Table 5 provides the information that the convergence processes in the rental market, compared to those in the sales secondary housing market, differ quite significantly. This may be due, as already mentioned above, to the very underdeveloped nature of the long-term residential rental market in Poland, which is additionally very much destabilised by short-term housing rents.

**Fig. 6:**
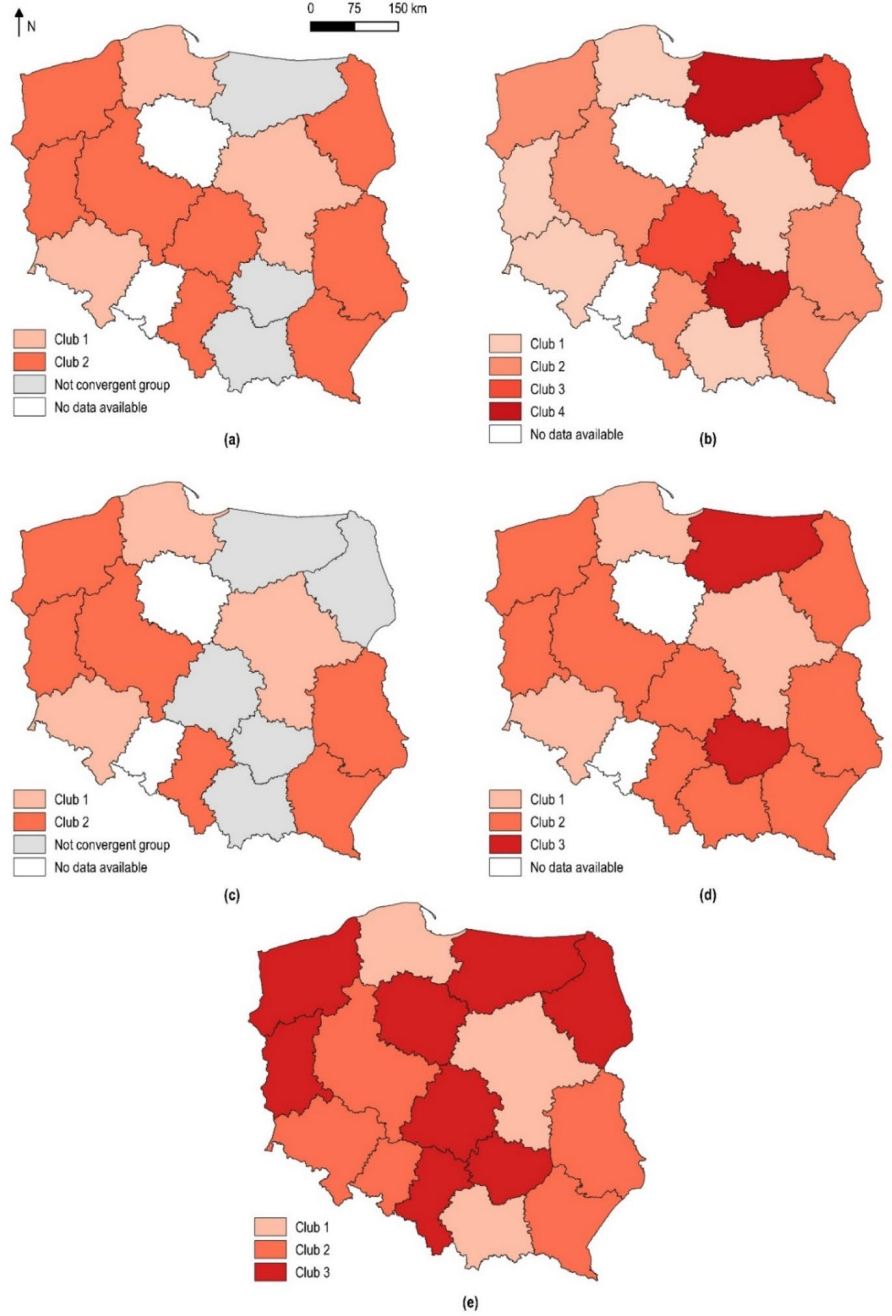
Spatial distribution of convergence clubs: in the Polish rental housing market identified based on the trend component of time series extracted using **a** the Hodrick–Prescott filter, **b** the boosted Hodrick–Prescott filter, **c** the Butterworth filter and **d** the Christiano–Fitzgerald filter; **e** in the Polish sales secondary housing market according to estimates by Tomal (2019a, b)

The geographical diversity of convergence clubs and divergent cases on the Polish residential rental market is very interesting. In particular, as can be seen in Fig. 6a–d, the allocation of individual cities to convergence clubs does not depend on their location. For example, cities included in the first clubs are located in opposite parts of Poland. On this basis, it can be concluded that spatial factors may not play a major role in the formation of convergence clubs.

**Fig. 7:**
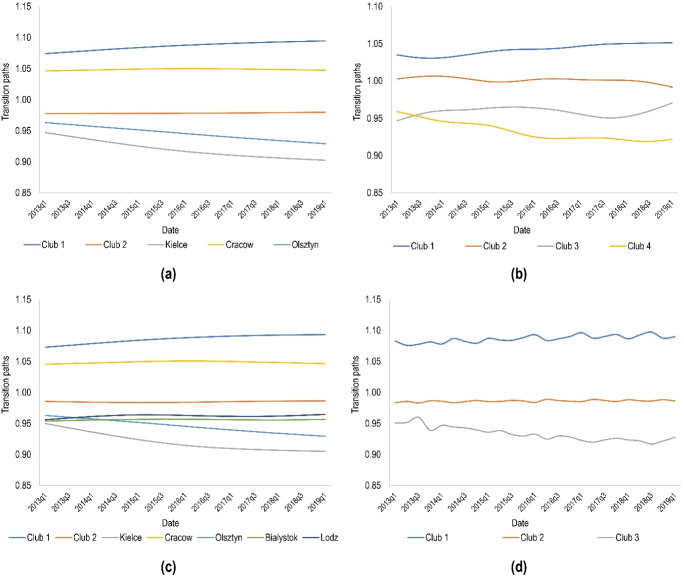
Transition paths for the clubs calculated based on the trend component of time series extracted using **a** the Hodrick–Prescott filter, **b** the boosted Hodrick–Prescott filter, **c** the Butterworth filter and **d** the Christiano–Fitzgerald filter

Another aspect worth examining during the cluster convergence analysis is transition paths for the clubs. As can be seen in Fig. 7, the identified clubs are unlikely to have a trend towards convergence. The only exceptions are the transition paths for clubs 2 and 3 in the case of data filtering with the boosted HP filter, where it can be assumed that in the following years these clubs may be merged and create a new city cluster.

**Fig. 8:**
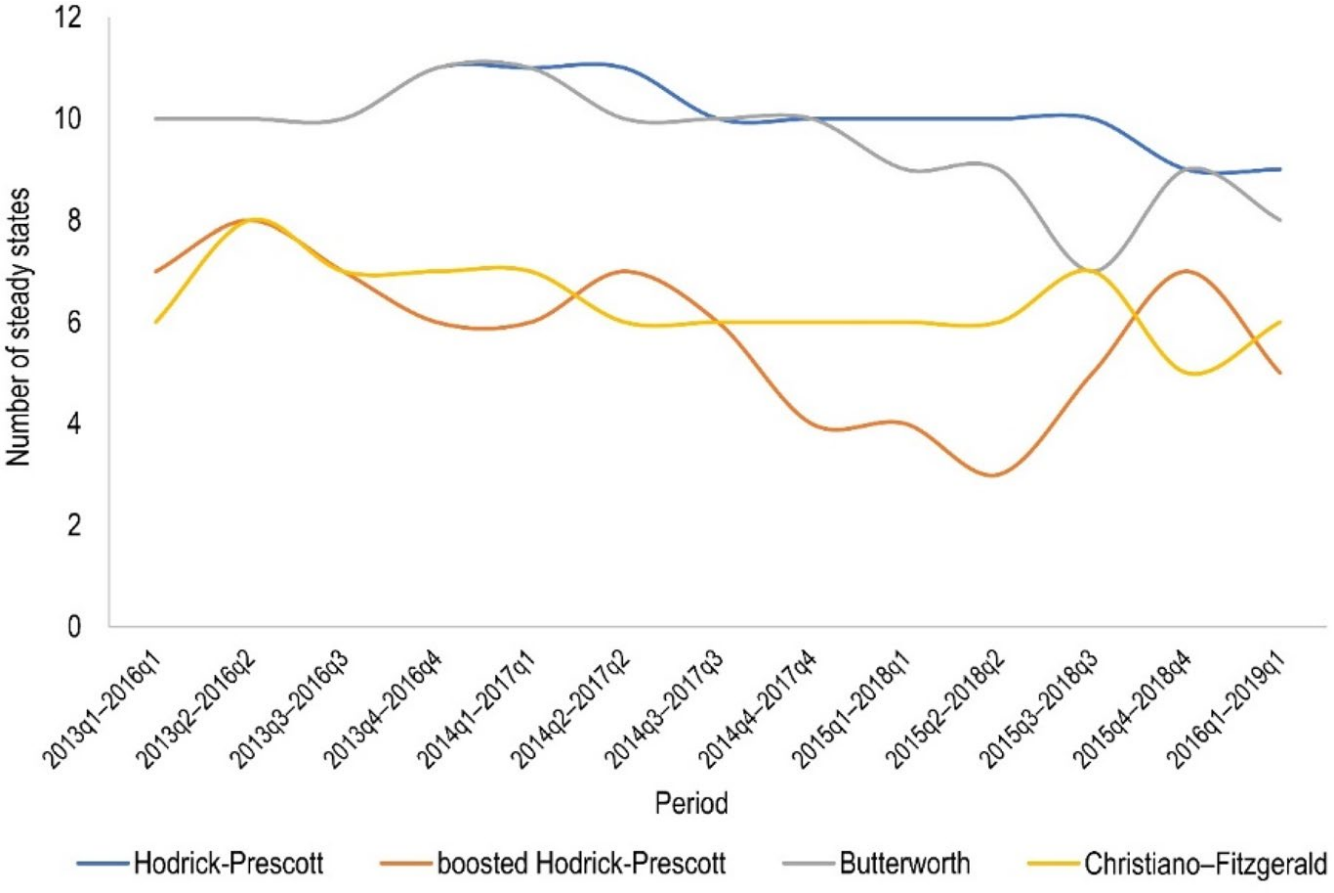
Number of steady states (convergence clubs plus divergent cases) in subsequent 3-year periods depending on the data filtering method

Finally, a dynamic club convergence analysis revealed that there was a slight decrease in the number of steady states in the surveyed lease market over the subsequent 3-year periods (Fig. 8). Furthermore, it can be seen that the time series obtained from the HP and Butterworth filters produce a very consistent number of steady states in the examined periods, which fluctuates around ten. The situation is also similar for the boosted HP and CF filters, but the number of long-term equilibria hovers around six.^10^ It can also be concluded from Fig. 8 that using the boosted HP filter, the changes in the number of steady states in the subsequent 3-year periods are the highest compared to other filters. The latter result may indicate that the boosted HP filter best reflects the dynamics of change in the real estate market in question, which is undoubtedly a great advantage when analysing rental or sales prices.

Summarising the results of cluster convergence testing on the Polish residential rental market, one can state that this market is highly fragmented, and the estimates obtained vary depending on the data filtering method. In particular, the club convergence analyses based on the trend components extracted using the HP, Butterworth and CF filters produce quite similar results (between 9 and 12 matches out of 14 cities in the whole analysed period). On the other hand, substantially different from the others, the estimates obtained from data filtered using the boosted HP method seem to be the most reliable.

### Robustness of cluster convergence estimates

In order to test the robustness of the results obtained in club convergence analysis, the approach outlined by Blanco et al. (2016) was applied. Specifically, it involves testing for the presence of stochastic convergence in the estimated convergence clubs. In other words, for each city in a given club, it is checked whether rental prices stochastically converge to the club average. Given that the speed of convergence in the defined clubs using the PS approach is small (for all cases $\hat{b} < 2$) and indicates convergence in growth rates, this robustness test will be performed in two specifications. The first one examines the stationarity of the variable $y_{itc} = {\text{ln}}p_{itc} - \overline{{{\text{ln}}p}}_{tc}$ where ${\text{ln}}p_{itc}$ denotes the log level rental price in city $i$ from club $c$ at time $t$ while $\overline{{{\text{ln}}p}}_{tc}$ refers to the average log level rental price at time $t$ in club $c$. The stationarity analysis of $y_{itc}$ allows to check whether rental price differentials between a given city and the club average tend towards zero or a nonzero constant. In the second robustness specification, a time series of the form $\Delta y_{itc}$^11^ is analysed to test whether growth rates are converging over time among members of a given convergence club. In the framework of Eq. (5) convergence in growth rates occurs when the estimated *t*-statistic for $\hat{\rho }_{i}$ is less than the critical value and simultaneously the fitted parameter $\hat{\alpha }_{i}$ is statistically insignificant.

Table 6 presents the results of the robustness tests conducted. Based on them, it can be concluded that there is no absolute price convergence in the identified convergence clubs, which is in line with the generated values of the parameter $b$ under the PS approach. Moreover, the test of stationarity of the variable $\Delta y_{itc}$ mostly confirmed the results obtained in the PS analysis showing convergence in the rates of change of rental prices. However, some differences can also be identified between the estimates generated by the PS approach and those by this robustness test. However, this applies only to the clustering based on the trend components obtained through the HP and Butterworth filters. For example, when analysing the estimates in Table 5 it can be seen that the cities of Kielce and Olsztyn are divergent units and are not part of any convergence club. However, the clusterings based on data filtered using the boosted HP and CF methods indicate that rental prices in the above-mentioned urban agglomerations form their own two-element convergence club. This fact has been confirmed by the robustness test carried out, which revealed that the growth rates of rental prices are equalising between these cities (see Table 6).

**Table 6:** Robustness tests results

Filter	Cities in a club	The time series analysed in terms of stationarity							
		$y_{itc}$				$\Delta y_{itc}$			
		$\hat{\rho }_{i}$	$\widehat{{{\text{CADF}}}}_{i}$	$\hat{\alpha }_{i}$	Conclusion	$\hat{\rho }_{i}$	$\widehat{{{\text{CADF}}}}_{i}$	$\hat{\alpha }_{i}$	Conclusion
HP, BW, CF	Gdansk	− 0.2583	− 1.8458	− 0.0201	Divergence	− 1.4961*	− 7.0527	0.0028	Convergence
	Warsaw	− 0.1212	− 0.9384	0.0211	Divergence	− 1.3527*	− 7.9277	− 0.0079	Convergence
	Wroclaw	− 0.5190	− 2.7305	− 0.0614	Divergence	− 1.4432*	− 8.2611	0.0056	Convergence
HP	Bialystok	− 1.0974*	− 4.8827	− 0.0834*	Convergence	− 1.6973*	− 10.3248	0.0024	Convergence
	Katowice	− 1.0148*	− 5.7005	0.0532	Convergence	− 1.4473*	− 10.0757	− 0.0049	Convergence
	Lublin	− 0.3365	− 1.4086	0.0432	Divergence	− 0.9095*	− 3.8035	− 0.0064	Convergence
	Lodz	− 0.6886	− 3.3993	− 0.0331	Divergence	− 1.3006*	− 5.9570	0.0070	Convergence
	Poznan	− 0.4152	− 2.1270	0.0594	Divergence	− 1.1572*	− 5.1937	− 0.0019	Convergence
	Rzeszow	− 0.5932	− 2.9938	0.0035	Divergence	− 1.1716*	− 5.0732	− 0.0039	Convergence
	Szczecin	− 0.3068	− 1.7752	0.0151	Divergence	− 1.3231*	− 6.5737	0.0038	Convergence
	Zielona Gora	− 0.3215	− 1.7753	− 0.0773	Divergence	− 1.2953*	− 5.9264	0.0058	Convergence
bHP	Cracow	− 0.6422	− 3.2433	0.0045	Divergence	− 1.1285*	− 5.9061	− 0.0098	Convergence
	Gdansk	− 0.3500	− 2.0212	0.0241	Divergence	− 1.2836*	− 5.8061	0.0025	Convergence
	Warsaw	− 0.0852	− 0.5790	0.0274	Divergence	− 1.5763*	− 6.5490	− 0.0066	Convergence
	Wroclaw	− 0.4245	− 2.3220	0.0187	Divergence	− 1.3496*	− 9.4841	0.0130	Convergence
	Zielona Gora	− 0.5737	− 2.7837	− 0.2659	Divergence	− 1.3744*	− 7.3163	− 0.0008	Convergence
bHP	Katowice	− 1.0578*	− 5.7005	− 0.0243	Convergence	− 1.2812*	− 7.8995	− 0.0049	Convergence
	Lublin	− 0.4134	− 2.2420	0.0210	Divergence	− 0.9374*	− 4.2950	− 0.0082	Convergence
	Poznan	− 0.6069*	− 3.4489	0.0429	Convergence	− 1.2192*	− 5.5802	− 0.0008	Convergence
	Rzeszow	− 0.8189*	− 3.6769	− 0.0540	Convergence	− 1.1543*	− 6.4070	0.0059	Convergence
	Szczecin	− 0.1737	− 0.9613	− 0.0019	Divergence	− 1.1530*	− 5.2608	0.0120	Convergence
bHP	Bialystok	− 0.9769*	− 4.5353	− 0.0121	Convergence	− 1.4881*	− 7.5612	− 0.0028	Convergence
	Lodz	− 0.9769*	− 4.5353	0.0121	Convergence	− 1.4881*	− 7.5612	0.0028	Convergence
bHP, CF	Kielce	− 0.6379	− 2.9675	− 0.0273	Divergence	− 1.5044*	− 7.8495	− 0.0009	Convergence
	Olsztyn	− 0.6379	− 2.9675	0.0273	Divergence	− 1.5044*	− 7.8495	0.0009	Convergence
BW	Katowice	− 0.8943*	− 4.7771	0.0355	Convergence	− 1.4098*	− 9.1846	− 0.0053	Convergence
	Lublin	− 0.3872	− 1.8142	0.0376	Divergence	− 0.8935*	− 3.8588	− 0.0047	Convergence
	Poznan	− 0.4736	− 2.4714	0.0618	Divergence	− 1.1844*	− 5.4726	0.0010	Convergence
	Rzeszow	− 0.6704	− 3.2060	− 0.0047	Divergence	− 1.3157*	− 6.4994	− 0.0035	Convergence
	Szczecin	− 0.3507	− 1.8517	0.0149	Divergence	− 1.3410*	− 6.4308	0.0054	Convergence
	Zielona Gora	− 0.2689	− 1.3495	− 0.0793	Divergence	− 1.3439*	− 5.9536	0.0058	Convergence
CF	Bialystok	− 1.0170*	− 4.3794	− 0.1030*	Convergence	− 1.6974*	− 10.3204	0.2591	Convergence
	Katowice	− 0.9795*	− 5.2588	0.0219	Convergence	− 1.4173*	− 8.7145	− 0.0024	Convergence
	Cracow	− 1.0129*	− 4.7988	0.2235	Convergence	− 1.4650*	− 7.4261	− 0.0013	Convergence
	Lublin	− 0.3209	− 1.3875	0.0301	Divergence	− 0.9847*	− 4.3688	− 0.0058	Convergence
	Lodz	− 0.6534	− 2.9376	− 0.0487	Divergence	− 1.1504*	− 5.3439	0.0053	Convergence
	Poznan	− 0.7433	− 3.3824	0.0854	Divergence	− 1.1947*	− 5.4920	− 0.0032	Convergence
	Rzeszow	− 0.5752	− 2.8386	− 0.0105	Divergence	− 1.2725*	− 6.0407	− 0.0046	Convergence
	Szczecin	− 0.3194	− 1.7984	0.0068	Divergence	− 1.3512*	− 6.3063	0.0029	Convergence
	Zielona Gora	− 0.4088	− 2.1468	− 0.1133	Divergence	− 1.2975*	− 7.6564	0.0060	Convergence

The convergence benchmark is the average log level rental price in a given convergence club. HP means Hodrick–Prescott, bHP boosted Hodrick–Prescott, BW Butterworth, and CF Christiano–Fitzgerald. The results of the study for diverging cities are not presented in this table due to the excessive number of possible cases. However, these results are available upon request

*Significant at 0.05 level

The robustness tests carried out, however, did not indicate which clustering is definitely the most reliable, i.e. whether the one obtained on the basis of data filtered with the boosted HP method or the one where the CF filter was applied. Therefore, in order to finally determine the most accurate division, the following formula was used^12^:

13 $$CP = \frac{{\mathop \sum \nolimits_{c = 1}^{C} \mathop \sum \nolimits_{i = 1}^{{N^{c} }} \mathop \sum \nolimits_{t = 1}^{T} \left| {\Delta y_{itc} } \right| }}{{\left( {T - 1} \right)\mathop \sum \nolimits_{c = 1}^{C} N^{c} }}$$

where $N^{c}$ denotes the number of cities in a convergence club $c$, while $\left| {\Delta y_{itc} } \right|$ should be interpreted as the absolute value of the difference between the growth rate in the city $i$ from club $c$ at time $t$ and the average growth rate in club $c$ at time $t$. The lower the CP value, the smaller, on average, the difference between the rental price growth rates in the cities and the average values in the clubs to which these cities belong, and consequently the estimated clubs are characterised by greater homogeneity.

The results indicated that the most accurate clustering was obtained by the PS approach, in which the boosted HP filter was used to extract the trend components of the time series. In particular, for this partitioning, the CP value was 0.0324, while for the clustering obtained when filtering the data with the CF method it was already 0.0339.^13^ Taking into account the robustness tests carried out and the results presented in Sect. 3.2, it should be concluded that the most reliable assignment of the examined cities to convergence clubs was proposed by the PS analysis, which used the trend components extracted using the boosted HP filter.

### What drives club convergence formation in the Polish rental housing market?

In order to check which factors, drive the formation of convergence clubs in the Polish residential rental market, a logit model was used, in which the dependent variable takes the value of 1 if two cities from a pair are in the same club, and 0 otherwise. It should be noted that in the case of the dependent variable defined in this way, the number of observations is 91 in the model, as the number of unique city pairs created is equal to $\frac{{N\left( {N - 1} \right)}}{2} = \frac{{14\left( {14 - 1} \right)}}{2} = 91.$

When examining aggregated prices in the housing market, as in this article, the explanatory variables are usually selected in the context of their impact on housing supply and demand. In terms of demand, the price determinants analysed are usually of demographic, economic and social nature. Conversely, supply variables describe, among others, the size of the housing stock. In this study, in order to select the covariates that may influence the formation of housing rental convergence clubs, the current scientific literature has been analysed, in which meso- and macro-determinants of sales housing prices on the Polish real estate market have been examined. Unfortunately, there are no empirical analyses explaining housing rental prices in Poland. Some studies, however, can be found at the micro-level, which concern tenants' preferences on local rental markets (see, for example, Gawlik et al. 2017).

The literature review indicated that the key predictors on the demand and supply side may be variables describing the level of unemployment (Leszczyński and Olszewski 2017; Tomal 2019a, b, 2021; Cellmer et al. 2020), wages (Cellmer et al. 2020), housing stock (Kokot 2018; Matysiak and Olszewski 2019; Tomal 2019b), population size and density (Tomal 2019a), city area (Tomal 2019a) and sense of security (Źróbek et al. 2015). The list of explanatory variables was supplemented with additional covariates characterising the specificity of the residential rental market in Poland, i.e. relating to tourist traffic (measured by total overnight stays) and the number of students in a given city. Moreover, the model contains regressors determining the distance between the surveyed cities and housing sales prices, in order to take into account spatial dependencies and links with the residential sales market, respectively. It should be stressed that since the dependent variable examines whether any two cities belong to the same convergence club, each regressor apart from the distance variable is defined as the absolute difference of the values for the cities in the pair.

The estimation results are presented in Table 7. In particular, models 1, 2, 3 and 4 were based on club convergence analysis estimates obtained from data filtered with the HP, boosted HP, Butterworth and CF methods, respectively. On the basis of the results obtained, it can be concluded that by far the best model in terms of goodness of fit is model 2 where the value of pseudo-*R*^2^ is 0.27 and is substantially higher than in the other models. Also, regarding the significance and direction of the impact of the average marginal effects, this model seems to be most in line with expectations. In particular, taking into account the factors affecting housing supply, it can be noted that the smaller the absolute difference in the number of dwellings and the area of a city in two cities the more likely it is that these cities will belong to the same convergence club. On the other hand, in terms of demand determinants, three variables are significant in model 2: population size, number of students and unemployment rate. For the latter two, the estimates of the average marginal effects are as expected, i.e. cities with similar unemployment rates and the number of students are more likely to belong to one convergence club. However, in the context of the population size variable, the situation is reversed. This may be due to the fact that in Poland the high level of development of the housing market is often not derived from the high population in a given city. For example, Cracow and Lodz, which are among the largest cities in Poland in terms of population, at the same time have opposite level of housing market development (see Fig. 1). The last variable that significantly shapes the formation of convergence clubs describes the distance between cities. The estimated parameter in model 2 indicates that the greater the distance, the greater the chance that any two cities will be in the same cluster. Paradoxically, taking into account the specificity of the Polish housing market, this is a desirable result. In particular, in Poland, the main urban agglomerations driving the development of the market under study are Cracow, Warsaw, Gdansk and Wroclaw. Analysing the location in space of these cities, one may notice that these local governments are located on opposite sides of Poland (see Fig. 6), and therefore, the estimated parameter for the distance variable seems plausible.

**Table 7:** Logit model estimates: drivers of convergence club formation

Variable	Average marginal effects							
	Model 1		Model 2		Model 3		Model 4	
Abs. dif. ln housing stock	− 1.63*	(0.87)	− 2.72***	(1.01)	− 2.79***	(1.02)	− 0.06	(1.16)
Abs. dif. ln population size	1.70*	(0.90)	2.79***	(1.03)	2.82***	(1.04)	0.04	(1.19)
Abs. dif. ln city area	− 0.26*	(0.14)	− 0.65***	(0.16)	− 0.41**	(0.16)	− 0.26	(0.17)
Abs. dif. ln crimes	0.30	(0.18)	0.66	(0.42)	0.49**	(0.21)	− 0.03	(0.23)
Abs. dif. ln total overnight stays	− 0.16	(0.10)	− 0.12	(0.11)	− 0.18*	(0.10)	0.18	(0.11)
Abs. dif. ln number of students	− 0.04	(0.10)	− 0.20*	(0.11)	− 0.06	(0.11)	− 0.08	(0.12)
Abs. dif. ln population density	0.29	(0.19)	0.02	(0.22)	0.22	(0.19)	0.55**	(0.24)
Abs. dif. ln housing prices	− 0.01	(0.44)	0.14	(0.49)	0.15	(0.47)	− 1.19**	(0.54)
Abs. dif. ln monthly wages	0.39	(0.52)	0.78	(0.55)	0.40	(0.54)	− 0.87	(0.67)
Abs. dif. ln unemployment rate	− 0.09	(0.15)	− 0.30*	(0.16)	− 0.08	(0.15)	0.34*	(0.17)
Ln distance	0.06	(0.10)	0.24**	(0.10)	0.13	(0.10)	0.03	(0.11)
Observations	91		91		91		91	
Pseudo-*R*^2^	0.16		0.27		0.20		0.15	

The dependent variable takes the value of 1 when any two cities belong to the same convergence club (0 otherwise). In models 1, 2, 3 and 4, the trend component of times series extracted using the Hodrick–Prescott filter, the boosted Hodrick–Prescott filter, the Butterworth filter and the Christiano–Fitzgerald filter were used, respectively. In parentheses, robust standard errors. Abs. Dif. means absolute difference

***Significant at 0.01 level

**Significant at 0.05 level

*Significant at 0.10 level

Examining the other estimated models (1, 3 and 4), it can be concluded that they have a rather low pseudo-*R*^2^ value, and moreover, the generated parameters for them are mostly insignificant as well as inconsistent with expectations; this applies, for example, to the parameter for the covariate describing the unemployment rate in model 4. Summarising the estimation results, one can notice that they fully confirmed the conclusions presented in Sects. 3.2–3.3 indicating that the most optimal tool for filtering the data under the PS approach is the boosted HP filter.

## Conclusions

In this article, an attempt was made to examine comprehensively the convergence of housing rental prices across Polish provincial capitals. In particular, this study aimed at answering the following research questions:

1. Do residential rents across the surveyed rental markets converge to one steady state in the long term? If not, can convergence clubs be identified among the cities studied?
2. In the case of absence of overall convergence in the Polish rental market, do club convergence analysis estimates differ depending on the time series filtering technique? Is the number of convergence clubs stable over time? Is the estimated structure of convergence clubs the same as in the case of the residential sales market?
3. In the case of absence of overall convergence in the Polish rental market, which factors drive the formation of identified convergence clubs?

Referring to question Q1, it should be stated that there is no overall convergence in the Polish residential rental market. It is possible, however, to identify convergence clubs where rental prices tend to move towards a club-specific steady state. In the scope of question Q2, club convergence analysis estimates indicate that, depending on the data filtering method, the division of cities into clusters differs significantly. Also, the dynamic analysis of steady states indicates that the number of long-term equilibria changes not only over time but also with the time series filtering technique. Moreover, on the basis of the revealed fragmentation of the residential rental market in Poland into convergence clubs, it can be concluded that it is not the same as that of the residential sales market. In the context of the final research question (Q3), a study using the logit model shows that cities with very similar unemployment rates, housing stocks, city areas and student numbers are very likely to be in the same convergence club.

This study obviously has some limitations. The first is the short time frame of the study, which is due to data availability. Another limitation is the lack of information for a larger number of localities, but this does not affect the results of the analysis to a large extent, as residential rents in Poland are concentrated mainly in major cities.

It should also be noted that this survey has revealed that the residential rental market in Poland is very fragmented. This may be a consequence of the underdeveloped nature of rental housing. Long-term residential leasing in Poland is also quite strongly destabilised by short-term renting, mainly for tourists, as well as by student tenancy. Therefore, state and local policy-makers should focus on the development of long-term renting as a supplement to home-ownership. This is necessary because of the huge housing shortages in Poland reaching up to three million units, as well as because of the increasing sales prices of housing, which exacerbates the residential affordability problem. One of the possible actions towards the development of the residential rental market in Poland is the promotion by policy-makers of institutional investments, for example, through land-use planning policy actions. In this context, as in the UK (see National Planning Policy Framework 2019), local politicians may consider introducing a mechanism for allocating certain locations to build-to-rent investment projects. A second possibility for politicians to promote tenancy is joint ventures of a public–private nature, in which a public entity could be a donor of land for future investments. Unfortunately, in Poland, the level of such activities is extremely low, and in the area of housing almost non-existent (Węgrzyn 2014; Tomal and Nalepka 2020). It should also be noted that renting in relation to purchasing is very often seen as an unprofitable form of providing a good such as housing (Ullah and Sepasgozar 2020). Therefore, policy-makers in Poland, through legislative actions, should solve the problem of artificially rising rents in the long-term market, which is a result of the growing stock of dwellings allocated for short-term tenancy.

Future research analysing the convergence processes in the Polish residential rental market should examine how the COVID-19 pandemic affected the behaviour of rental prices between the examined locations. It may be assumed that due to the lockdown of the entire economy in Poland, which was introduced several times, the processes responsible for the formation of the ripple effect in the residential market have weakened considerably. In particular, this may affect the rental market to a much greater extent than the sales market, which is more dependent on population, labour market and financial capital mobility.

## Data Availability

The data used in the survey were obtained from the websites of the National Bank of Poland (https://www.nbp.pl/) and the Local Data Bank (https://bdl.stat.gov.pl/BDL/start).

## Footnotes

1 As aptly noted by Ganioğlu and Seven (2021), the literature on overall and cluster convergence does not emphasise the difference between the use of house/rental prices in real or nominal terms. A similar view is also taken by Holmes et al. (2017). Therefore, this study prefers the use of nominal prices because they exhibit more spatiotemporal variation. Furthermore, this will allow to juxtapose the results obtained with the study carried out by Tomal (2019a) for the residential sales market, which was done on nominal prices. However, for comparison purposes, the whole convergence study was also carried out using real prices and the results obtained did not show significant differences to those presented in this paper. The estimates of this additional analysis are available upon request.

2 The lag truncation parameter must be determined in order to estimate the *t*-statistic (and in particular the long-run variance) for the parameter $\phi$ in Eq. (2). More details are available in the paper by Kong et al. (2019).

3 Following Carvalho and Harvey (2005) a time trend is not included in the CADF regression because it is inconsistent with a convergence model and it makes ADF-based tests less powerful.

4 Phillips and Sul (2007) proved by simulation that the size distortion in the case of small *T* (especially when $T = 10$) occurs only when the convergence rate parameter $\alpha$ is close to zero. Moreover, the distortion disappears very quickly when *T* or $\alpha$ increases. A simulation by Phillips and Sul (2007) also showed that the proposed convergence test is characterised by high power for small samples (the exception being the case when $T = 10$). Du (2017) performed additional simulations to test the effectiveness of the clustering algorithm proposed by Phillips and Sul (2007). In general, the results obtained indicated that the false discovery rate is acceptable, even for small samples, i.e. when $T = 20$. Considering the above, the PS approach is a suitable tool for overall and cluster convergence analysis for short time series analysis.

5 Derivation of the log *t* regression equation from the factor model presented in Eq. (6) and the semiparametric representation in Eq. (7) is discussed in detail in Appendix B of the Phillips and Sul (2007) paper.

6 According to a simulation by Phillips and Sul (2007) the setting for small samples of $r = 0.3$ is the best choice, as this allows both to secure size accuracy in the test and also does not reduce its power excessively.

7 There are other methods available in the literature for identifying convergence clubs, which in particular are based on the concept of stochastic convergence (see, for example, Hobijn and Franses 2000; Corrado et al. 2005; Pesaran 2007b). However, as Apergis and Payne (2017) the PS approach outperforms club convergence methods using unit root or cointegration tests.

8 For more on the disadvantages of the CF filter, see the work by Iacobucci and Noullez (2005).

9 The correlation coefficient between the *t*-statistics obtained on the basis of the data filtered using the HP and Butterworth methods is 0.98 and is statistically significant. For the other estimates, the correlation coefficients oscillate between 0.46 and 0.55 and are also statistically significant.

10 When analysing the dynamics of change in the number of steady states, similar to the estimated *t*-statistics presented in Fig. 5, the highest correlation is between the results obtained based on the trend components extracted using the HP and Butterworth filters. In this case, the correlation coefficient is equal to 0.63. For remaining estimates, the correlation coefficients range from 0.11 to 0.38.

11 The expression $\Delta y_{itc}$ can also be written as $\ln \left( \frac {p_{itc}} {p_{{itc}-1}} \right) - \ln \left( \frac {{\overline p}_{tc}} {{\overline p}_{{tc}-1}} \right)$ which is equivalent to the difference between the growth rate in the city *i* from club *c* at time *t* and the average growth rate in club *c* at time *t*. It should be stressed that the study of the equalization of growth rates by means of stochastic convergence is not frequent, however, it is nevertheless possible to present here some works (see, for example, Kočenda, 2001).

12 The conceptualised CP indicator is only applicable if convergence in growth rates takes place in the estimated clubs.

13 When dividing the cities into clubs based on data filtered using the HP and Butterworth methods, the CP values were 0.0353 and 0.0327, respectively. However, these results are not fully comparable with those where data were filtered using the boosted HP and CF filters, as the latter assigned all cities to clubs, while the former resulted in diverging units as well.

## Code availability

The identification of convergence clubs was performed based on the Stata module by Du (2017). Also, the estimation of logit models was conducted using the Stata software, in particular the logit command. Furthermore, the R package by Phillips and Shi (2020) was used to extract the trend components of the time series using the boosted Hodrick–Prescott method. Finally, to assess σ-convergence the Stata module by Kong et al. (2019) was applied. The rest of the calculations were carried out in MS Excel.
